# Nonlinear spectral tunability of pulsed fiber laser with semiconductor optical amplifier

**DOI:** 10.1038/s41598-022-17796-7

**Published:** 2022-08-13

**Authors:** Anastasia Bednyakova, Daria Khudozhitkova, Sergei Turitsyn

**Affiliations:** 1 Novosibirsk State University, 1 Pirogova str., Novosibirsk, 630090 Russia; 2 Aston Institute of Photonic Technologies, Aston University, Birmingham, B4 7ET UK

**Keywords:** Nonlinear optics, Optics and photonics, Fibre lasers, Mode-locked lasers

## Abstract

We examine spectral properties of radiation in the pulsed fiber lasers using the semiconductor optical amplifier (SOA) as the gain medium. The complex light dynamics that result from the interplay between the fiber propagation effects in the cavity, the nonlinear effects in the SOA and spectral filtering, shift the generated radiation from the central wavelength of the filter. The resulting wavelength of the output radiation depends on the SOA pump power and the bandwidth of the intracavity filter. This offers the possibility of a spectral tunability of the generated pulses through nonlinear dynamics rather than the conventional use of a tunable filter.

## Introduction

The properties of a range of modern lasers are defined by the nontrivial light dynamics introduced by the nonlinear effects in the resonator. Nonlinearity can be both distributed along the cavity (e.g. Kerr effect, or nonlinear polarisation rotation in the optical fiber) or produced by a point-like action of some elements (e.g. saturable absorber or nonlinear amplifier with a scale small compared to the length of resonator). Nonlinear effects, while not easy to control, might offer rich possibilities for developing a variety of new laser sources^1–11^.

SOA is a well-established practical optical device with numerous attractive properties including compact size, wide gain bandwidth and the possibility of direct gain modulation by controlling the injection current. SOAs are important in a wide range of applications including optical signal processing^12^, wavelength division multiplexing (WDM), optical time division multiplexing (OTDM)^13^, wavelength switching^14^, optical clock recovery^15,16^, low-noise, high-bit rate optical sampling^17^ and others.

Moreover, SOA is also promising for applications beyond traditional optical communications and semiconductor lasers. For instance, it can be used as a gain medium in fiber lasers instead of rare-earth doped active fibers. To the best of our knowledge, the first publications on SOA-based lasers started to appear in the late 70 s^18–25^. Matsumoto and Kumabe^18^ have demonstrated the AlGaAs-GaAs ring lasers with different three-dimensional waveguide structures, including pillbox type and a circle type. An optical-fiber composite-cavity lasers, semiconductor-optical fiber ring lasers including mode-locked lasers have been studied in the pioneering works^19–22^. Theory for a linewidth of a semiconductor-optical-fiber ring laser was developed in^26^ by using coupled rate equations for the active and passive cavity. SOA-based mode-locked lasers can generate ultra-short pulses of the order of hundreds of femtoseconds^27^. Mode-locking was achieved by an external optical non-return-to-zero data injection. In the ring-cavity fiber laser^28^ sub-picosecond optical pulses were generated, where nonlinear polarization evolution in SOA served as a mode-locking mechanism. In^29^, a mode-locked laser with a ring cavity is presented, which generates pulses that can be compressed down to 274 fs in an external compressor. Hech et al. demonstrated a possibility of generating pulses with the pulse duration down to 300 fs at 1550 nm in the mode-locked semiconductor ring laser^30^. The scheme includes SOA and a saturable absorber based on the InP/InGaAsP technology as well as passive components providing frequency dispersion. Nyushkov et al. demonstrated fiber laser, mode-locked via SOA modulation with injection current pulses and controllable shape of generating light pulses^31,32^. Figure-of-eight SOA-based fiber lasers were investigated in^33,34^. It was observed in^34^ that the pulse repetition rate depends on the injection current of the SOA almost linearly and can vary over a wide range from 30 MHz to 12.02 GHz. A self-starting passively harmonic mode-locked laser producing pulse train with near half duty-cycle at a repetition rate of 1.7 GHz was demonstrated in^33^. A combination of fiber resonator with SOA to generate pulsed radiation also offers an interesting possibility of designing systems based on nonlinear dynamics of light.

Recently we demonstrated both numerically and experimentally^35^, that nonlinear properties of a SOA can be used to shift the central wavelength of input pulses to a blue part of the spectrum, opposite to the well-known Raman-induced red-shift^36,37^. In this work we examine nonlinear spectral shifts (from the central wavelength of an in-cavity filter) of pulses generated in a fiber laser system using SOA. In the considered SOA-based fiber laser, SOA plays the role of both gain element and a nonlinear pulse transformer. We numerically determine cavity parameters leading to a single-pulse generation and analyse the dependence of the spectral tunability around the central wavelength of the filter on the filter bandwidth, cavity dispersion and the gain of SOA.

## Scheme of a fiber laser

**Figure 1:**
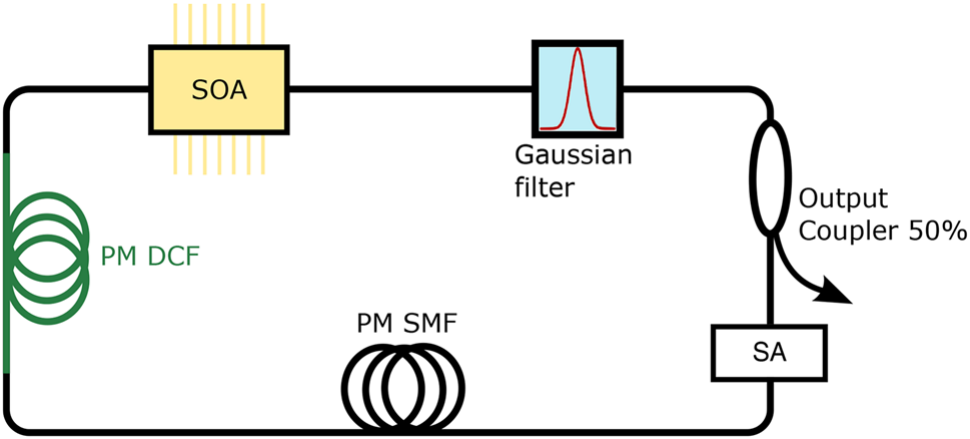
Schematics of a SOA-based fiber laser setup.

Figure 1 shows the schematic depiction of the fiber laser setup we are studying. The laser cavity is comprised of polarization-maintaining dispersion-compensating fiber (PM DCF), polarization maintaining single-mode fiber (PM SMF), a semiconductor optical amplifier (SOA), a saturable absorber (SA) element, an output coupler and a spectral filter with 1550 nm central wavelength. The length of SMF was an optimization parameter that allows us to vary the cavity dispersion from normal to anomalous to change the pulse propagation regimes. The filter bandwidth was also considered as an optimization parameter, while the parameters of SOA and saturable absorber were fixed.

Pulse propagation down the fiber spans is governed by the standard nonlinear Schr$\ddot{o}$dinger equation (NLSE) with the loss term, all details of numerical modeling for all cavity elements including DCF, SMF, SOA and the saturable absorber are given in the “Methods” section. We first consider a simplified model that does not include gain dependence on the wavelength inasmuch as spectrum width of the laser pulses is much smaller than the typical SOA gain bandwidth. We start from this model to demonstrate the key effects and will consider the impact of the spectral gain profile on pulse formation in the separate section. An output coupler, placed after Gaussian filter, takes 50% of the radiation power from the cavity.

## Pulse evolution without spectral filtering

First, we recall key features of lasing in SOA-based fiber laser without spectral filtering of radiation. When we remove the Gaussian spectral filter from the laser cavity it is also possible to find conditions for a single pulse generation. We define single pulse generation to be established when a relative variation of pulse energy, peak power and pulse width does not exceed 0.1% during the previous 20 round trips. The cumulative dispersion of the cavity varied in the range from 0.5 to − 0.5 ps$^2$ via corresponding SMF lengthening from 0 to 50 meters, while the piece of DCF was fixed at 5 meters.

**Figure 2:**
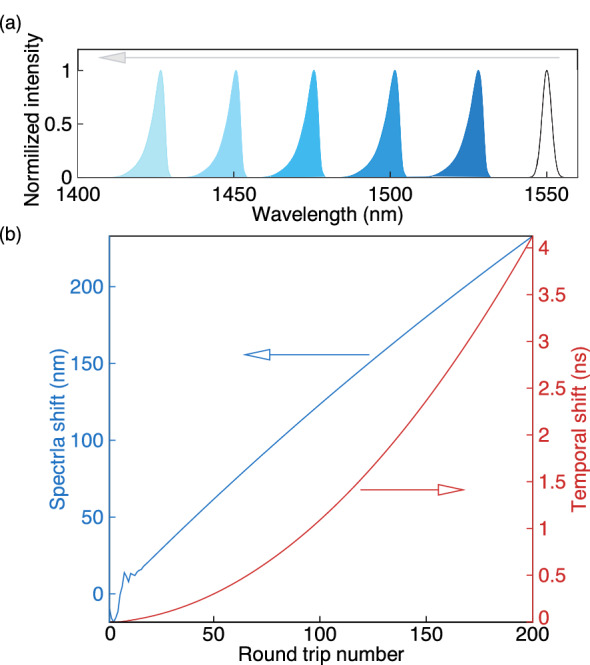
(**a**) Shape of the intracavity spectrum after SOA depicted at every 20th round trip of the cavity. Black line shows the initial spectrum. (**b**) Evolution of peak power position and central wavelength of the output pulse during the first 200 round trips. Here the laser cavity includes 5 meters of DCF and 35 meters of SMF. No spectral filter is used.

It was observed, that in the case of anomalous cavity dispersion $\beta _2^{cum} < 0$ the above-mentioned pulse parameters can be stabilized. However, the center frequency of the pulse is continuously shifted to the blue side of the spectrum due to the negative chirp acquired in the anomalous-dispersion cavity (Fig. 2a,b)^35^. The shape of the spectral power distribution of the pulse does not change during this continuous spectral shifting. The pulse is accelerated in the time domain with propagation (Fig. 2b) and with its temporal position constantly shifting. Note that a similar soliton dynamics was studied in a long-distance fiber transmission with semiconductor amplifiers^38–40^.

The observed pulse evolution with unlimited continuous wavelength shift is due to the simplified model assuming an infinite gain bandwidth. Therefore, a more realistic model should either take into account a finite gain bandwidth or stabilise this continuous spectral shift by spectral filtering. In the next section a Gaussian filter is added to the cavity to stabilize single-pulse generation.

## Pulse stabilisation by the spectral filtering

In the previous section, we have observed that a pulse generated in the SOA-based fiber laser (without spectral filter) continuously changes its temporal position and central wavelength at the cavity output. Moreover, one can see that in the considered set of system parameters, a generated pulse demonstrates a continuous spectral blue shift. This spectral sliding dynamics can be stabilised by a filter. Without loss of generality, we consider a Gaussian-shaped filter with the variable bandwidth placed after SOA. The central wavelength of the filter was at 1550 nm, the bandwidth varied from 2 to 50 nanometers.

**Figure 3:**
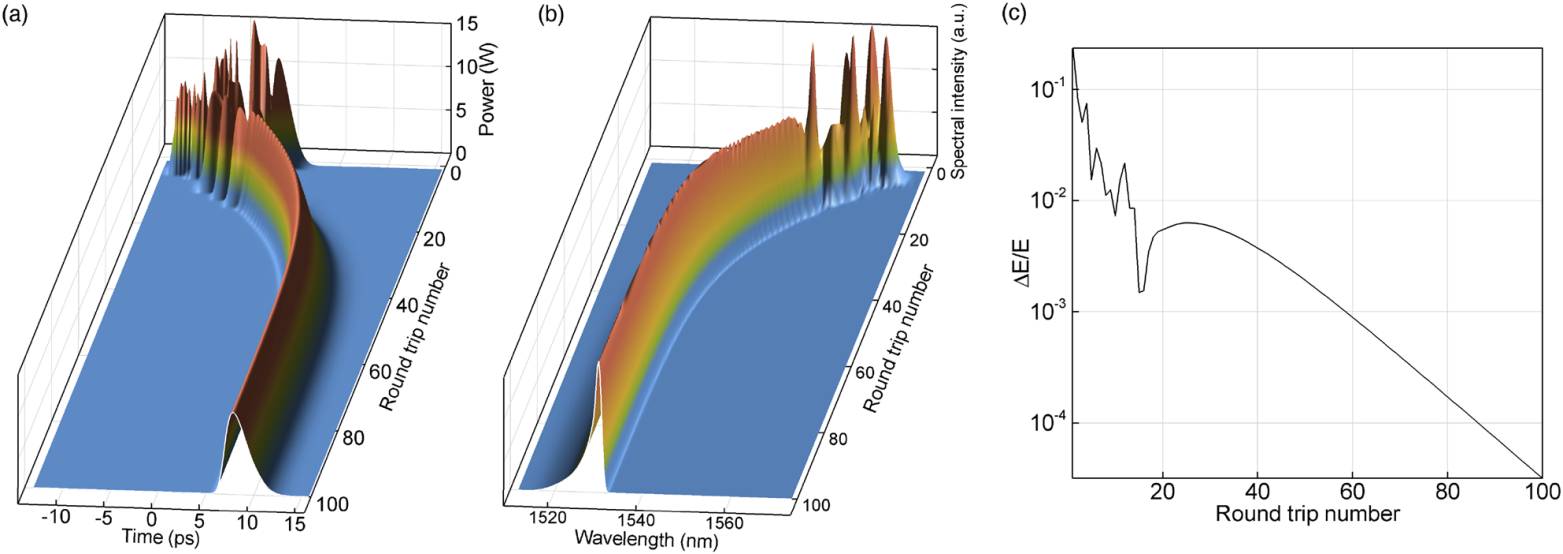
Evolution of pulse power (**a**) and power spectral density (**b**) with round trips in SOA-based fiber laser with 34 nm Gaussian filter. (**c**) Convergence of the output pulse energy variation to zero during the first 100 cavity round trips.

It is seen that spectral filtering leads to the stabilization of the pulse spectrum and temporal shape (Fig. 3a,b). Note, that both pulse spectrum and temporal shape are asymmetrical due to the SOA transient response. The regime of single-pulse generation converges to a steady state after approximately 40-50 cavity round trips. Figure 3c shows convergence of the relative energy difference calculated over the two consecutive round trips $\Delta E/E = |E_{i+1}-E_{i}|/E_{i} \xrightarrow [i \rightarrow \infty ]{} 0$, where *i* is the round trip number.

**Figure 4:**
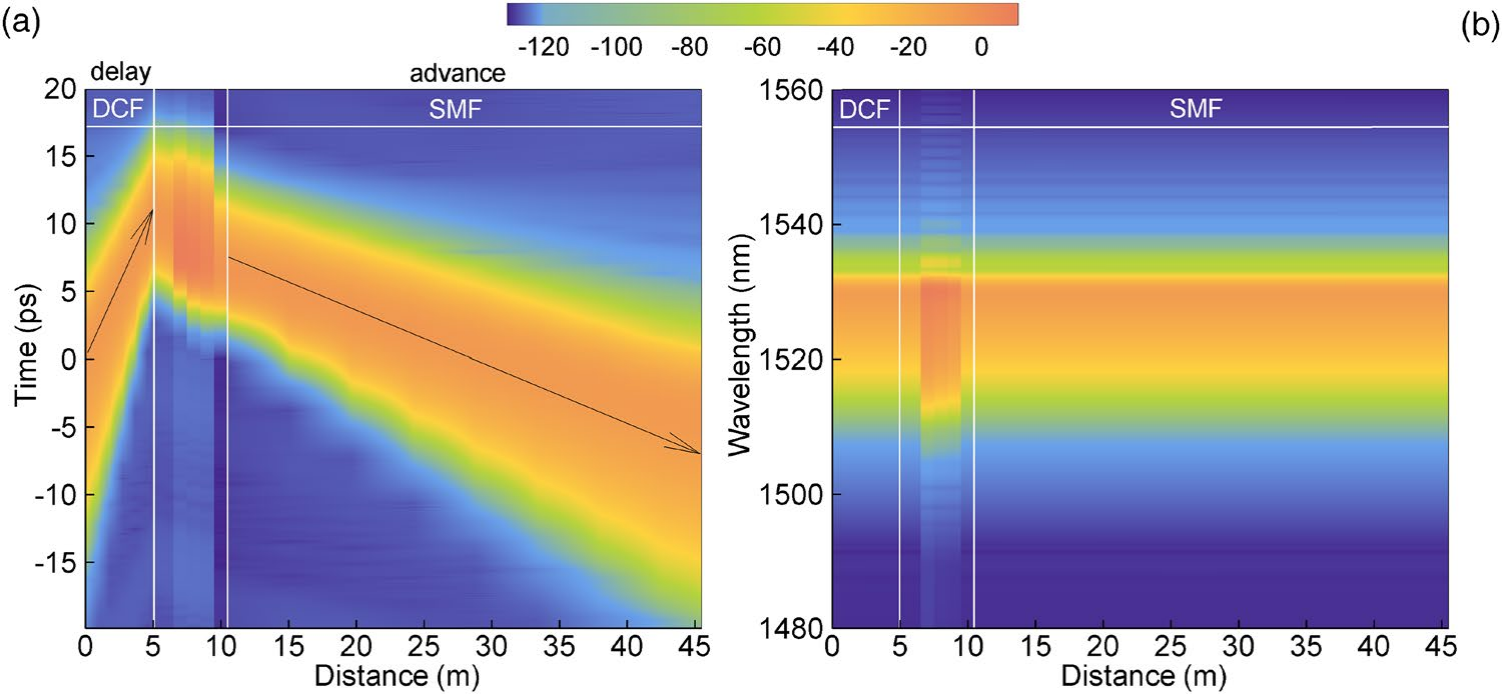
Evolution of the pulse power (**a**) and power spectral density (**b**) along the fiber sections.

Intracavity evolution of the pulse, shown in Fig. 4 has several characteristic features. It is seen that the pulse group velocity alternates along the resonator. The group velocity changes sign moving from DCF to SMF due to the nonlinear frequency chirp acquired after amplification (Fig. 4a). Tunable temporal shift of the chirped pulses in SOA-based system was previously demonstrated and studied in^41^. At the same time, the central wavelength of the spectral power density (Fig. 4b) does not change along the fiber sections.

**Figure 5:**
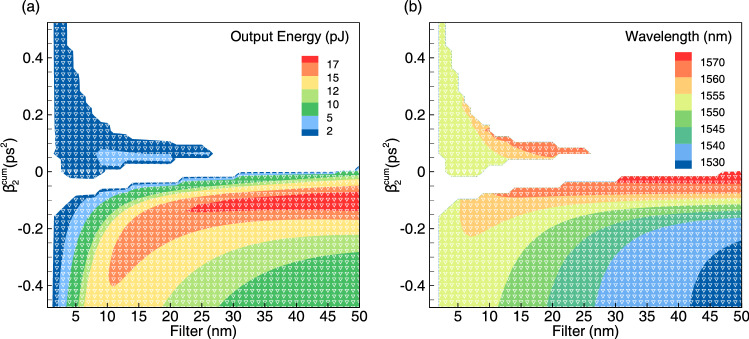
Output pulse energy (**a**) and central wavelength (**b**) as functions of the filter bandwidth and cumulative cavity dispersion.

A map of the steady-state single pulse regimes is shown in Fig. 5. Here, filter bandwidth and cumulative cavity dispersion are considered as variable parameters. The energy (Fig. 5a) and the central wavelength (Fig. 5b) of the output pulses are depicted by color. We can conclude that the anomalous cavity dispersion offers more flexibility for single pulse generation. It is seen that in the case of anomalous cavity dispersion the central wavelength of the output laser pulse shifts from red to blue part of the spectrum with an increase in the bandwidth of the spectral filter. When the cavity dispersion is normal (positive), single pulse generation is also possible, however, in a relatively small area and under the condition of strong spectral filtering. In what follows, we consider the case of the anomalous dispersion in more detail, as it enables stable generation and wavelength control of the output pulse.

**Figure 6:**
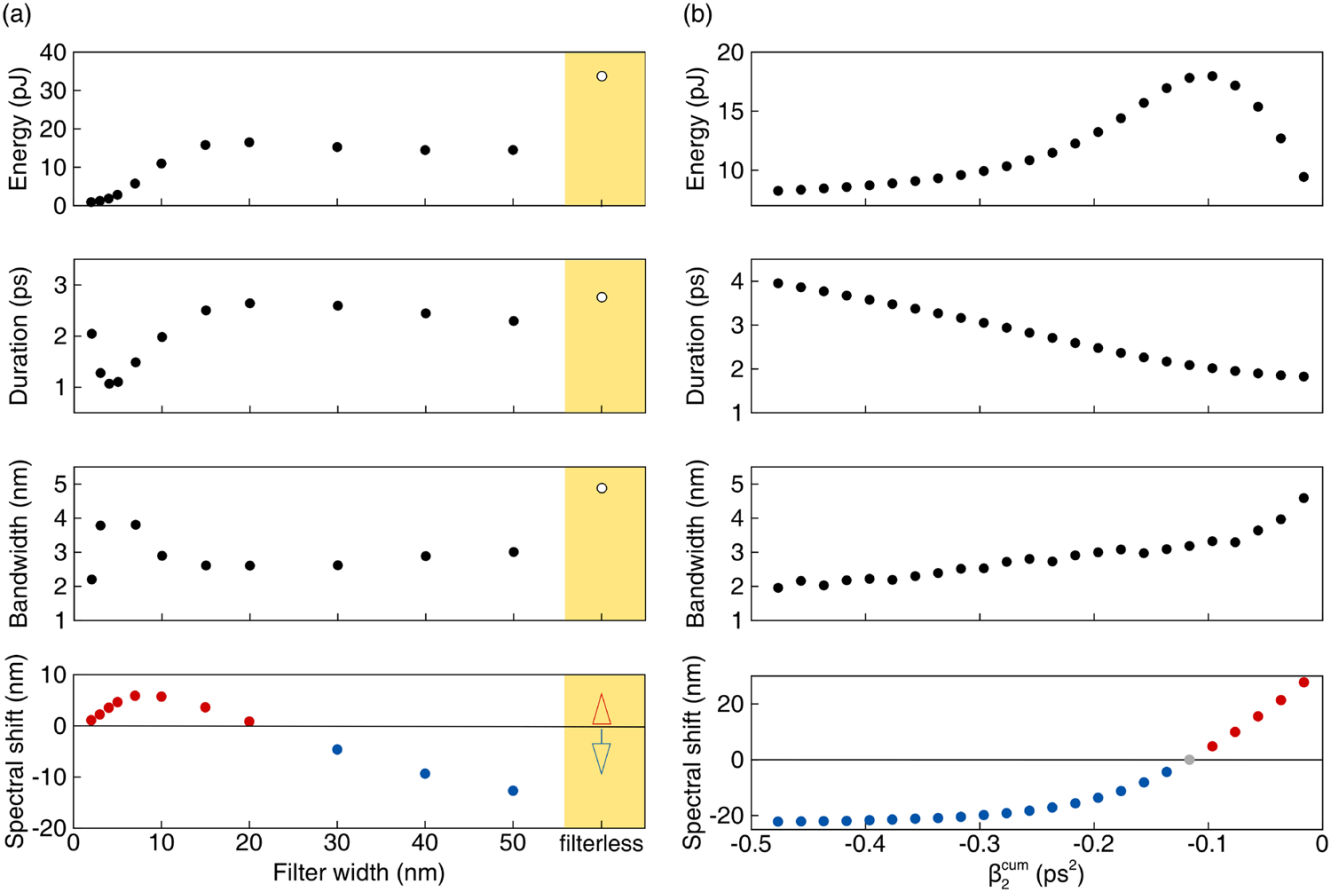
Dependence of energy, duration, bandwidth and spectral shift of the intracavity pulse on: (**a**) the filter bandwidth (the cavity dispersion is $\beta _2^{cum} = - 0.18$ ps$^2$); and on (**b**) the cumulative dispersion (filter bandwidth is 45 nm). Empty circles correspond to the laser cavity without filter.

Figure 6a shows how the main pulse characteristics (energy, spectral bandwidth, temporal duration and center wavelength) depend on the filter width at a fixed cavity dispersion. Here, the cavity dispersion is $\beta _2^{cum} = - 0.18$ ps$^2$ (35-meters long SMF). Empty circles depict the output pulse characteristics in the cavity without the spectral filter. The pulse central wavelength shifts from − 13 to 6 nanometers relative to the central wavelength of the filter. For example, a 50 nm filter results in the blue spectral shift of 13 nm. The wider the filter bandwidth, the stronger the spectral shift.

Figure 6b demonstrates how the cavity dispersion affects the generated pulse parameters. Here, the filter bandwidth is fixed and equal to 45 nm. As expected, the pulse duration decreases as the dispersion leans towards zero. The pulse bandwidth increases accordingly. It is seen that a blue shift of the central pulse wavelength changes to red in the region of small anomalous dispersion. The point with the maximum 28 nm red shift corresponds to the laser cavity comprising of 5-meters long DCF and 27-meters long SMF.

**Figure 7:**
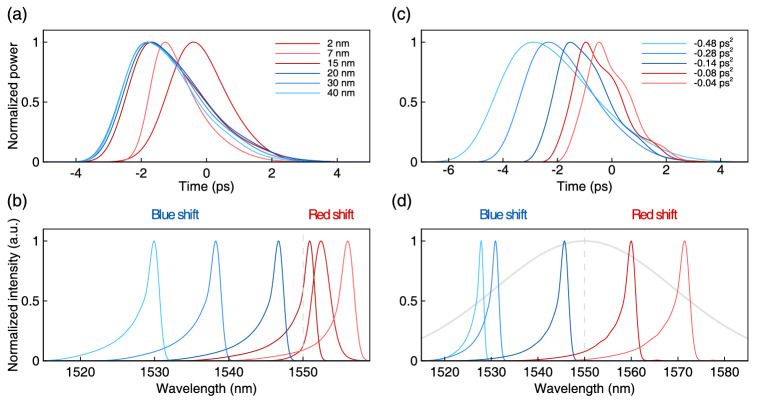
Temporal power distribution (**a**) and spectral power density (**b**) of the laser pulses, corresponding to spectral filters with varying bandwidth (from 2 to 50 nm) and fixed 1550 nm central wavelength (dashed gray line). Temporal power distribution (**c**) and spectral power density (**d**) of the laser pulses corresponding to varying cumulative dispersion of the laser cavity (from − 0.04 to − 0.4 ps$^{2}$). The Gaussian spectral filter (45 nm) centered at 1550 nm is shown by the gray line.

Spectral and temporal shapes of the generated pulses corresponding to different filter bandwidths at the fixed cavity dispersion are shown in Fig. 7a,b. The pulse power spectral density and power for varying dispersion (with the fixed filter bandwidth) are presented in Fig. 7c,d.

## Impact of a finite gain bandwidth

The bandwidth of standard SOAs can be in a wide range starting from  5 THz (40 nm)^42^. Recently developed SOA structures based on quantum dots (QD-SOA) made it possible to significantly expand the gain spectrum up to 90-120 nm^43,44^. Moreover, in^45^ the InGaAs/AlAs solution-processed QD-SOA using superimposed quantum structure has been proposed for development of ultra-broadband SOA. Changing the number of quantum dots groups made it possible to increase the bandwidth up to 1.02 μm.

**Figure 8:**
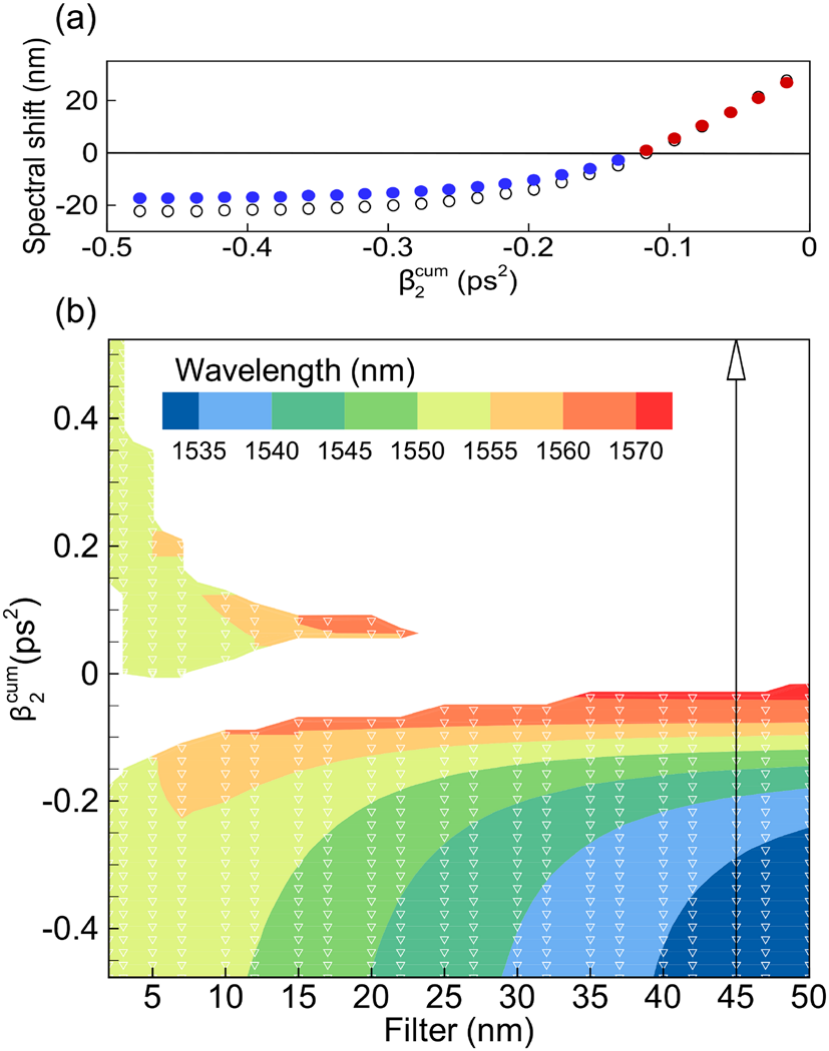
(**a**) Shift of the wavelength of the generated pulse from the central wavelength of the optical filter for varying cumulative dispersion (here filter bandwidth is 45 nm). Empty circles correspond to the laser with the infinite gain bandwidth. (**b**) Output pulse wavelength as a function of the filter bandwidth and cumulative cavity dispersion.

Above we have considered the SOA-based fiber laser with infinite gain bandwidth. In this section, we verify the validity of these results by examining a more realistic case that takes into account the gain bandwidth limitation. Gain profile used in simulations has a Lorentzian shape^46^ with 200 nm width at half maximum and 1550 nm central wavelength. Laser setup used in simulations is the same as in the previous sections. Filter bandwidth and cavity cumulative dispersion are also considered as variable parameters. Dependence of the output pulse central wavelength on dispersion and filter bandwidth is shown in Fig. 8. Figure 8a depicts how the spectral shift of the output pulse, calculated at 45 nm filter, changes for finite gain bandwidth in comparison with infinite gain case (empty circles correspond to infinite gain). It can be seen that a blue shift becomes 20% smaller for a fixed 45 nm Gaussian filter, while a red shift remains practically unchanged. Also the full map of the steady-state regimes, shown in Fig. 8b, looks similar to the infinite gain bandwidth case (Fig. 5b). The largest shift obtained in simulations is 20 nm towards the blue part of the spectrum and 28 nm towards the red part.

Thus, when the 200-nm gain bandwidth is taken into account in the numerical model, the nonlinear pulse dynamics does not change significantly, while the range of spectral tuning becomes   20% smaller. It is important to take into account gain bandwidth more accurately when the range of the spectral tuning is close to the gain bandwidth.

## Control of the spectral tunability of the generated pulses by SOA gain

In many lasers a central frequency of the bandpass optical filter defines the spectral position of the peak of the spectral power density of the generated radiation. As we observed in previous sections, the central wavelength of the generated pulses in the considered laser system is defined not simply by the spectral position of an optical filter, but also by the nonlinear dynamics of radiation in the cavity. The central wavelength of pulses can be shifted by changing either cavity dispersion or filter bandwidth. Although a possibility to tune the central wavelength of the radiation by changing cavity parameters affecting nonlinear light dynamics is interesting, the flexible adjustment of dispersion or filter bandwidth is not that straightforward and easy implementable in efficient manner. Therefore, in this section we explore a more practical opportunity of controlling the central wavelength of the output pulse by varying the SOA pump power (or in terms of our model by changing small-signal gain parameter $h_0$). We vary the small signal gain $h_0$ in the range from 15 to 30 dB, for three values of the cumulative dispersion ($\beta _2^{cum} = -0.017, -0.18, -0.48$ ps$^2$) and observe the spectral properties of the resulting pulse generation. Here, the Gaussian filter parameters are fixed with the central wavelength of 1550 nm and a bandwidth of 40 nm.

**Figure 9:**
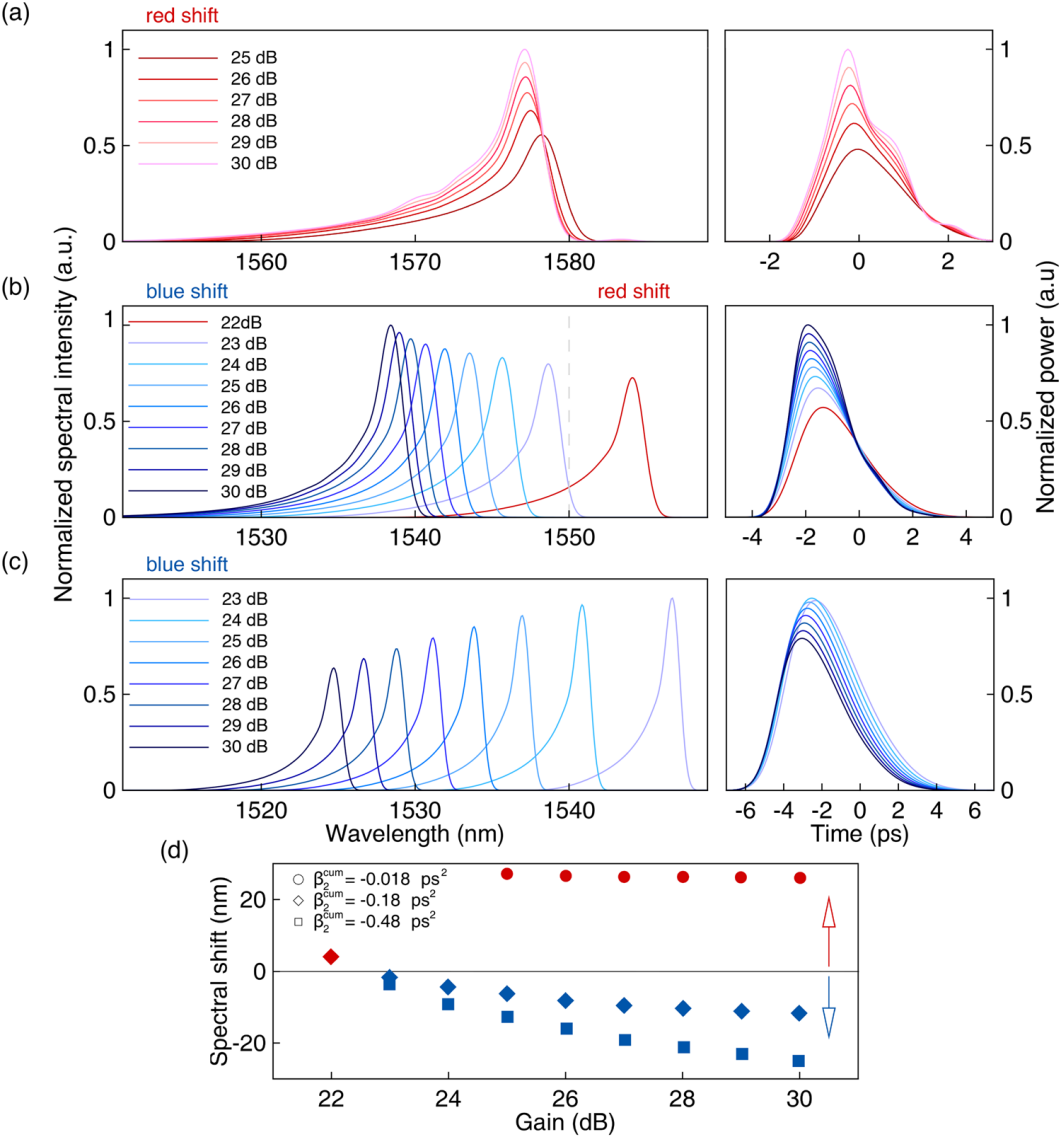
Spectral (left) and temporal (right) profiles of the steady-state pulses for different cavity dispersion and small-signal gain (marked on the figures): (**a**) $\beta _2^{cum} = -0.017$ ps$^2$, (**b**) $\beta _2^{cum} = -0.18$ ps$^2$, (**c**) $\beta _2^{cum} = -0.48$ ps$^2$. (**d**) Spectral shift of the generated pulse from the central wavelength of the optical filter for varying small-signal gain parameter $h_0$.

Figure 9 shows the spectral (left) and temporal (right) profiles of the steady-state pulses corresponding to: $\beta _2^{cum} = -0.017$ ps$^2$ (a), $\beta _2^{cum} = -0.18$ ps$^2$ (b) and $\beta _2^{cum} = -0.48$ ps$^2$ (c). When the cumulative dispersion is small and close to zero ($\beta _2^{cum} = -0.017$ ps$^2$), stable pulsed generation in the laser occurs at $h_0 \ge 25$ dB. Variation of the small-signal gain (pump power) does not lead to large changes in the position of the spectrum. The peak of the spectrum is red-shifted relative to the filter central wavelength (1550  nm) by approximately 28 nm at the point of the maximum shift.

In the case of $\beta _2^{cum} = -0.18$ ps$^2$, pulse generation starts from $h_0=22$ dB. We observe spectral shifts (again, relative to 1550  nm) from 4 nm to the long-wavelength region at $h_0=22$ dB and up to 11.5 nm to the short-wavelength region at $h_0 = 30$ dB. Thus, changing the pump power makes it possible to vary the central wavelength of the generated pulses in the range of about 15 nm in this case, implementing both red and blue shifts relative to the filter central wavelength. In the third case we consider a sufficiently large cumulative cavity dispersion of $\beta _2^{cum} = -0.48$ ps$^2$. The generation threshold in this case is $h_0 = 23$ dB. We observed in this case spectral blue shifts (from 1550  nm) ranging from 3 nm at $h_0 = 23$ dB to 25 nm at $h_0 = 30$ dB, offer an overall tunability of around 20 nm. Note, that in the case $\beta _2^{cum} = -0.48$ ps$^2$ the increase of the pump power leads to a decrease of the pulse energy due to spectral filtering induced losses. It is clear that by further optimizing the cavity parameters or using special designing laser resonators, we can extend tunability implemented in this rather practical way—by changing the SOA driving current.

## Conclusion

We demonstrate that nonlinear effects in the fiber laser with a SOA offer the possibility of the spectral tuning of the generated pulses. The underlying nonlinear dynamics are based on the interplay between the Kerr nonlinearity (self-phase modulation) induced pulse spectral broadening and the inherent SOA power-to-phase conversion of the modulated radiation. These nonlinear dynamics lead to a shift in the wavelength of the generated pulses relative to the central wavelength of the in-cavity filter (1550  nm in the considered example). Spectral shift can be both to the red or blue side of the spectrum depending on the bandwidth of the filter and cumulative cavity dispersion. Most interestingly, we demonstrate that the wavelength of the generated pulse can be shifted by adjusting the SOA pump power (small-signal gain).

## Methods

Numerical modelling of the intra-cavity light dynamics has been implemented by a consecutive computation of the field evolution in the laser elements.

### Light evolution in the fibre spans

Pulse propagation down the fiber spans is governed by the standard nonlinear Schr$\ddot{o}$dinger equation (NLSE) with the loss term:

1 $$\begin{aligned} \frac{d A}{d z}=-i\frac{\beta _{2}}{2}\frac{\partial ^{2}A}{\partial ^{2} t^2}+i\gamma |A(z,t)|^2 A(z,t)-\frac{\alpha }{2}A(z,t), \end{aligned}$$

where *A*(*z*, *t*) is the slowly-varying amplitude of the pulse envelope, *z* is the propagation coordinate, *t* is the time, $\beta _{2}$ is the group-velocity dispersion (GVD), $\gamma$ is the Kerr nonlinearity coefficient, and $\alpha$ is the fiber loss parameter. The values of the fiber parameters: $\beta _{2} = 127.5$ ps$^{2}$/km, $\gamma = 4.66$ (W km)$^{-1}$, $\alpha = 0.4$ dB/km for the DCF, and $\beta _{2} = -20$ ps$^{2}$/km, $\gamma = 1.3$ (W km)$^{-1}$, $\alpha = 0.18$ dB/km for the SMF.

### Light transformation in SOA

The conventional model of SOA includes modifications of the power *P* and phase $\phi$ of the optical field $A= \sqrt{P}\, \exp ( i \phi )$ by the amplification, and a differential equation for the time-dependent gain *h*(*t*)^42^:

2 $$\begin{aligned} P_{out}(t)&= P_{in}(t) \exp [h(t)], \nonumber \\ \phi _{out}(t)&=\phi _{in}(t)-\frac{1}{2}\alpha _{H}h(t),\nonumber \\ \frac{d h}{d t}&=-\frac{h-h_0}{T_{SOA}}-\frac{P_{in}(t)}{E_{sat}}\left[ \exp (h)-1\right] , \end{aligned}$$

where $P_{in/out}(t)$ is the input/output power, $\phi _{in/out}(t)$ is the input/output phase of the optical signal, $\alpha _{H}$ is the linewidth enhancement factor, $h_0$ is the integral small-signal gain, $T_{SOA}$ the gain recovery time, $E_{sat}$ is a characteristic saturation energy. We consider without loss of generality the following typical parameters: $\alpha _H=4$, $E_{sat}=6$ pJ, $T_{SOA} = 200$ ps and $h_0 = 27$ dB for all numerical modeling hereinafter. Note that this model does not include gain dependence on the wavelength inasmuch as spectrum width of the laser pulses is much smaller than the typical SOA gain bandwidth. We start from this model to demonstrate the key effects and will consider the impact of the spectral gain profile on pulse formation in a separate section.

### Transfer function of the saturable absorber

The saturable absorber element nonlinear response is modelled by the standard general equation:

3 $$\begin{aligned} |A_{out}(t)|^2= (1-\frac{\alpha _{0}}{1+\frac{|A_{in}(t)|^2}{P_{SA}}}-\alpha _{ns} ) \; |A_{in}(t)|^2, \end{aligned}$$

where $\alpha _{0} = 0.36$ is the saturable absorption, $\alpha _{ns} = 0.64$ is a non-saturable loss parameter, $P_{SA} = 10$ W is the saturation power.

## Data Availability

The datasets used and/or analysed during the current study are available from the corresponding author on reasonable request.
